# Assessment of event-based vision sensor cameras for measuring bubbly flow characteristics

**DOI:** 10.1007/s00348-026-04272-5

**Published:** 2026-06-30

**Authors:** Ahmed Al Brahim, Kuppuraj Rajamanickam, A. M. K. P. Taylor, Yannis Hardalupas

**Affiliations:** 1 Department of Mechanical Engineering, Imperial College London, London, SW7 2AZ UK; 2 Department of Mechanical and Aerospace Engineering, The University of Manchester, Manchester, M13 9PL UK

## Abstract

In this study, the capability of Event-based Vision Sensor (EVS) cameras for measuring simultaneously bubble motion, size, and shape in complex bubbly flows has been assessed. This assessment initially focused on benchmark evaluation of the accuracy of an EVS camera in tracking the motion and size of precisely manufactured naturally buoyant particles in quiescent water. Experiments were carried out in synchronization with a conventional high-speed (HS) camera, enabling quantitative comparison. The synchronized measurements obtained from both cameras agreed well with differences of less than 0.65% in measured particle velocities and 2.4% in measured diameters, demonstrating the EVS camera’s capability to precisely measure motion and dimensions. Following this validation, the assessment expanded to characterize bubbles generated by injecting compressed air at various pressures at the bottom of a quiescent water tank. The EVS camera demonstrated comparable performance to the HS camera in measuring bubble size and velocity at low to moderate injection pressures. The bubble tracks uniquely captured by the EVS camera, due to its adjustable accumulation time, can be leveraged to extract trajectory and velocity information, as well as instances of partial bubble visual overlap in the camera view. However, event saturation is observed at the EVS camera for several events generated by bubble motion that surpasses a critical threshold (e.g., dense bubbly flow). Subsequently, the increased latency at both pixel levels and read-out leads to temporal misalignment relative to the flow bubble dynamics. This challenge was addressed by employing a pulsed illumination source, which allowed the camera to capture dense bubbly flows, as the increased presence of bubbles corresponds to a reduced number of EVS recorded events. Overall, the assessment highlights that EVS cameras offer comparable performance to conventional HS cameras in characterising bubbly flows with two additional unique advantages. (i) Ability to record bubble tracks, which can be directly used for bubble velocimetry. (ii) An instantaneous EVS camera image records events at a significantly reduced data rate, which enables real-time bubble EVS imaging at higher speeds than conventional HS cameras.

**Supplementary Information:**

The online version contains supplementary material available at 10.1007/s00348-026-04272-5.

## Introduction

Bubbly flow is prevalent across a wide range of natural phenomena and industrial processes, including chemical reactors (Schlüter et al. 2021), water electrolysis (Kempler et al. 2024), boiling heat transfer (Ghazivini et al. 2022), and water treatment (Levitsky et al. 2022). Developing a comprehensive understanding of bubble dynamics in complex flows is vital for improving the performance and control of these processes. However, the inherent complexity resulting from multiscale interactions between gas bubbles and the surrounding liquid poses significant challenges to achieving a comprehensive understanding. These interactions lead to rapid changes in bubble motion, shape, and interface. Consequently, analytical solutions are generally restricted to highly idealized cases, while numerical simulations are computationally demanding and often rely on approximations. These approximations can limit their ability to fully capture the underlying complex physics involved and necessitate the validation using experimental datasets (Besagni et al. 2023). In addition to validating analytical and numerical simulations, experimental techniques could provide accurate measurements and a new understanding of bubbly flows.

Among the key experimental techniques, shadowgraphy is widely used for visualizing bubble morphologies and their interactions (Al-Behadili et al. 2023), (Tan et al. 2023), (Wang et al. 2024). Traditionally, frame-based cameras are used for shadow imaging of bubbles. Despite their effectiveness, these cameras can be costly and constrained by a limited recording duration, especially for high-speed imaging. Extended recording at a high frame rate is crucial for investigating transient bubbly flow phenomena, for example, bubble characterization near the critical current density during water electrolysis (Park et al. 2024), and the correlation of bubble behaviour with the pool boiling curve in thermal systems (Liang and Mudawar 2019). In contrast to conventional High-Speed (HS) cameras, Event-Based Vision Sensor (EVS) cameras have recently emerged as a promising alternative to overcome temporal sampling limitations, thanks to their reduced data rate.

**Fig. 1:**
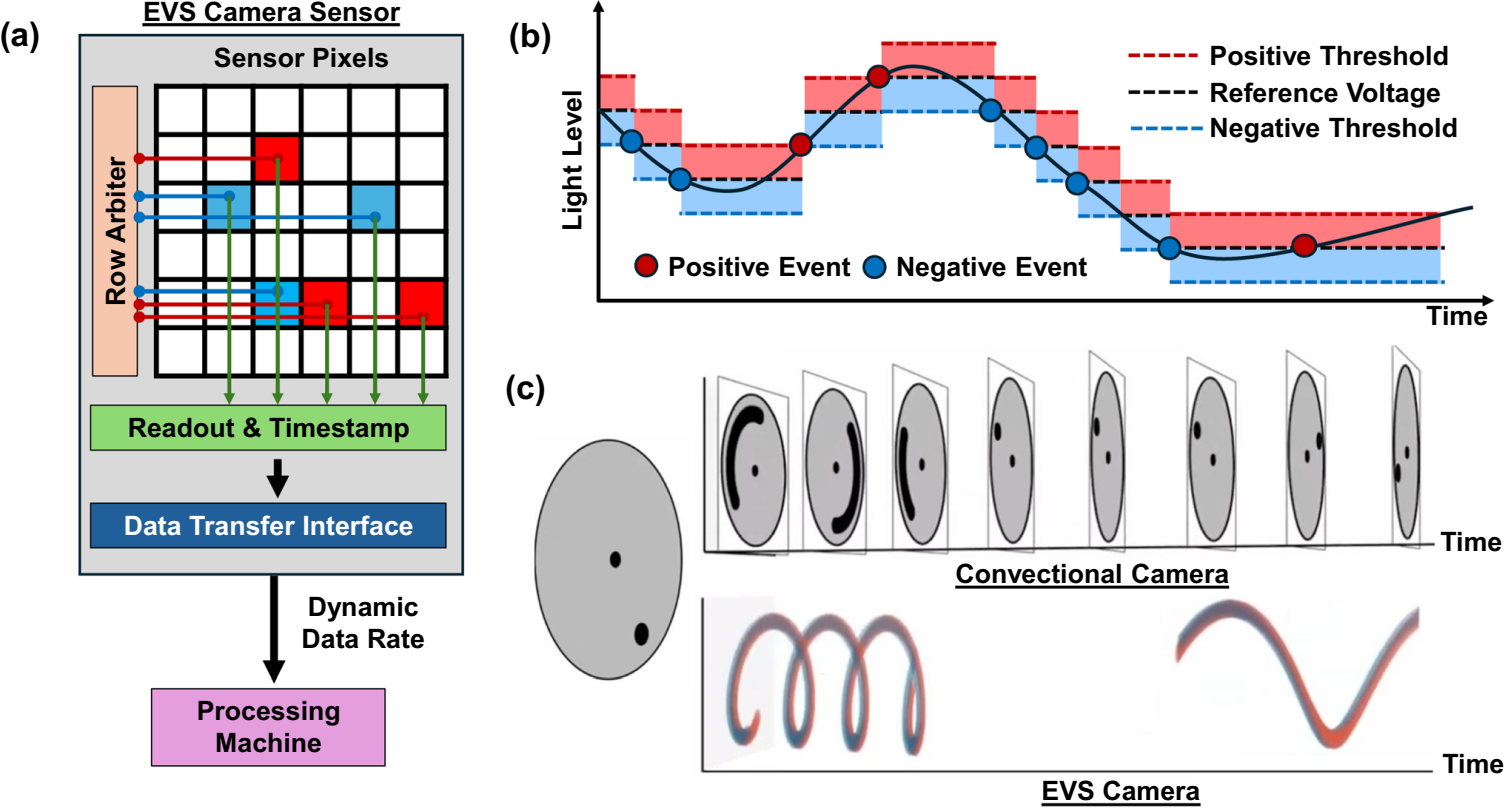
**a** Typical architecture of an EVS camera. **b** Event generation mechanism, where changes in light intensity above or below set thresholds trigger positive or negative events, respectively. **c** Comparison of recordings of a rotating disk marked with a dot, captured by both a conventional frame-based camera and an EVS camera, adapted from Bull and Zhang (Bull and Zhang 2021)

Inspired by the human retina (Mahowald 1992), EVS cameras detect motion through asynchronous pixels that only respond to changes in brightness. As depicted in Fig. 1 (a), these pixels operate independently and generate discrete positive (ON) or negative (OFF) events once the brightness change exceeds a predefined threshold, as illustrated in Fig. 1 (b). In contrast to frame-based cameras that capture entire scenes at fixed intervals, event cameras continuously record pixels that detect changes in light intensity. A row arbiter selectively scans rows containing activated pixels, allowing the events to be read and time-stamped by the processing unit. Each registered event contains four pieces of information: the pixel’s x and y coordinates, time of occurrence, and polarity (positive or negative). The working principles of EVS cameras are explained in more detail in the work of (Rebecq et al. 2021).

The difference between an EVS camera and a conventional frame-based camera output can be demonstrated by capturing a rotating disk marked with a dot along its perimeter, as shown in Fig. 1 (c). The frame-based camera captures images at fixed intervals, recording both the disk and its static background. In contrast, the EVS camera continuously registers the motion of the dot over time, without recording any background information, as the background remains unchanged. When the disk stops rotating, the frame-based camera continues to capture identical frames while the EVS produces no events due to the absence of motion. Furthermore, if the disk rotates at high speed, the frame-based camera output may suffer from motion blur. In contrast, the EVS remains unaffected due to its asynchronous, motion-triggered sensing mechanism, which can precisely provide the dot’s position in real time. Consequently, EVS cameras offer substantial reductions in data redundancy, motion blur, and power consumption by transmitting information exclusively in response to dynamic changes in the scene. The recorded events are represented using two colours corresponding to event polarity (i.e., positive/negative), resulting in considerably smaller data volumes than those produced by frame-based cameras. This feature facilitates the direct transfer of event data to a connected computer, enabling extended recording durations limited solely by the host computer’s storage. Whereas high data rates associated with traditional HS cameras necessitate temporary storage of data in RAM, limiting the recording duration.

Despite the advantages of the EVS camera, its latency in recording events poses a serious drawback in many applications. Here, latency refers to the time taken by the pixel to respond to a change in light intensity (pixel latency) and the delay incurred in the timestamping of the detected events during the readout process (readout latency), as depicted in Fig. 1 (a). The latency is typically very low and in the order of a few microseconds (Gehrig and Scaramuzza 2024). However, due to the continuous recording of events, when multiple pixels are activated simultaneously, an increase in the number of events will introduce latency at both pixel and readout levels. Such latency eventually leads to a mismatch between the time stamping and the actual timing of the real-world event. Hence, limiting the number and rate of activated pixels is required to avoid significant latency.

Advancements in EVS camera technology have significantly improved recording speeds, dynamic range, latency, and pixel resolution. Modern EVS cameras can achieve the same high frame-rate recordings as conventional frame-based systems while maintaining high dynamic range and minimal latency (Kryjak 2024). Additionally, they are typically compact in size, which enables them to be placed in small spaces. Furthermore, modern EVS cameras are comparatively less expensive than most conventional high-speed cameras. Due to the EVS camera’s features, its capabilities have been demonstrated in diverse applications, including automotive (Chen et al. 2020; Shariff et al. 2024), vibration monitoring and diagnostics (Li et al. 2024) and, fluid mechanics. In fluid mechanics studies, the EVS camera capabilities have been demonstrated for particle tracking velocimetry (PTV) of pipe flow (Drazen et al. 2011), and aerodynamic flows (Borer et al. 2017), Particle Image Velocimetry (PIV) in turbulent flows (Raffel et al. 2024; Franceschelli et al. 2025), and spray applications (Rajamanickam and Hardalupas 2025). The use of pulsed illumination has been introduced for particle tracking to enhance the EVS camera’s handling of slow-moving particles and to prevent the EVS row arbiter latency from affecting the results (Willert 2023).

To explore the potential of an EVS camera in bubbly flow applications, this study aims to expand the assessment of EVS cameras in measuring bubble size and velocity in bubbly flows. First, the canonical case of naturally buoyant and precisely manufactured spherical particles is considered to compare the performance of EVS to a frame-based HS camera in particle sizing and velocimetry, thereby providing a reliable benchmark. Following validation, synchronized measurements using conventional HS and EVS were carried out in bubbly flows with varying bubble densities. Experiments were also performed using both continuous and pulse illumination; their performance, capabilities, and limitations in the context of bubbly flow characterization are meticulously compared, thereby providing guidelines for optimal utilization of EVS systems.

## Experimental methodology

**Fig. 2:**
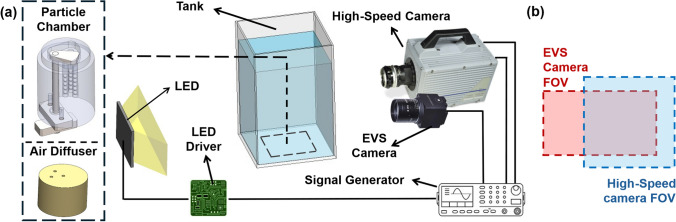
**a** Schematic diagram outlining the experimental configurations for synchronized imaging of bubbly flow and particle release by EVS and HS cameras. **b** The overlapping FOV region between the cameras is used for the analysis

As illustrated in Fig. 2 (a), the experimental setup comprises an EVS camera, a frame-based HS camera, a signal generator, a Light-Emitting Diode (LED) driver, a high-power LED, and a quiescent water tank that can be fitted with a particle chamber or an air diffuser. The tested EVS camera is the PROPHESEE EVK4, featuring a maximum resolution of 1280 × 720 pixels, a dynamic range exceeding 120 dB, a low light cutoff of 0.08 lx, and a pixel latency of less than 100 µs. This camera’s frame rate can surpass the equivalent of 10,000 fps in conventional frame-based cameras. The performance of the EVS camera is compared to the Photron APX-RS HS camera (full sensor resolution: 1024 × 1024 pixels) through synchronized experiments. Both cameras use identical 50 mm lenses for minimal distortion, with the aperture set to f/11 to ensure sufficient depth of field and image sharpness. Furthermore, small aperture settings are required in EVS cameras to suppress random events caused by ambient light, as they are highly sensitive to small changes in light intensity.

Image mapping between the EVS and the HS camera was achieved by using a ruler image. We were able to place an event camera adjacent to the HS camera without an angular offset, thanks to its small size. However, as shown in Fig. 2 (b), the horizontal distance covered by the two cameras differed. Thus, only the overlapped area between the two cameras is considered during the analysis. Restricting the analysis to the overlapped area, together with the common-path refraction through the flat tank interface, significantly reduces optical and geometric distortions in relative measurements. It is worth noting that more accurate spatial mapping could have been achieved using the calibration target (checkerboard/dot pattern). However, this requires a transparent (glass/acrylic) target plate with pulsed illumination to enable image capturing by an event camera. To evaluate the effect of magnification on measurement performance, experiments were conducted at different spatial resolutions by varying the camera distance from the particle release and bubble generation locations and by using extension rings. The corresponding magnification scales are described in later sections.

A function generator (TTi tgp110) is used to trigger the high-speed camera, and the same signal is fed into the EVS data stream to synchronize both cameras. This step is essential, since the EVS camera acquires data in continuous mode, requiring a well-defined external common trigger signal to synchronize the discrete frames of the HS camera with the EVS camera output. The frame rate of the HS camera was set to 1000 fps. Back illumination required for shadow imaging is provided by an in-house developed high-power LED system. In this study, the performance of the EVS camera is evaluated under both continuous and pulsed illumination conditions. For continuous illumination, the LED is directly connected to the DC power supply, while pulsed illumination is achieved through a MOSFET-based LED driver. The light pulsing frequency was set to 1000 Hz (pulse width: 500 ns), synchronizing with the HS camera frame rate. Both the light pulsing frequency and pulse width have a significant influence on the EVS camera performance, which will be explained in greater detail in the relevant sections.

A transparent tank filled with water, measuring 10 × 10 × 10 cm, is placed between the cameras and the LED. The bottom of the tank is equipped with either a particle chamber or an air diffuser to introduce particles or bubbles into the quiescent water. The particle chamber slots have a diameter of 4 mm and are loaded with Cospheric polypropylene polymer particles, which are naturally buoyant in water and have a diameter of 2.483 mm ± 0.004 mm and a density of 0.9 g/cc. The difference in slot size and the particle size is intended to prevent the simultaneous release of multiple particles and the jamming of the particles in the slots. After loading the particles, the chamber slots are filled with water and inspected to ensure that there are no bubbles in the slot that could block the particle release or reside on them. Before filling the tank with water, the particle chamber slots are closed with a gate that is manually opened to facilitate the release of particles.

Bubbles were produced with a 0.3 mm air diffuser connected to an adjustable air supply at the tank bottom. Experiments were conducted with varying bubble diameters, generation rates (f_b_), and velocities by adjusting the air flow rate (Q). In some experiments, a surfactant was added to reduce the water surface tension (σ) to 25.9 mN/m, helping to form more spherical bubbles. Table 1 summarizes the experimental cases considered.

**Table 1:** Experimental cases for bubbly flow generation at different compressed air flow rates and surface tensions, with the resulting bubble generation frequencies

Case	Q (ml/s)	σ (mN/m)	f_b_ (Hz)
I	6	25.9	4.65
II	75	70.1	100
III	200	70.1	250
IV	800	25.9	375

The tested EVS camera settings (e.g., intensity threshold, sensor noise level) can be fine-tuned for optimized recording. Before recording, the camera bias settings were modified to limit events caused by background noise. When using pulsed illumination, the threshold for detecting decreases in light intensity (OFF threshold), as highlighted in Fig. 1 (b), is set to a high value to suppress the generation of negative events, as they could overload the camera with unusable events. Furthermore, the refractory period is tuned to define the post-event duration of pixel blindness, ensuring that each significant intensity change, induced by a light pulse, triggers only one event in the activated pixels.

**Fig. 3:**
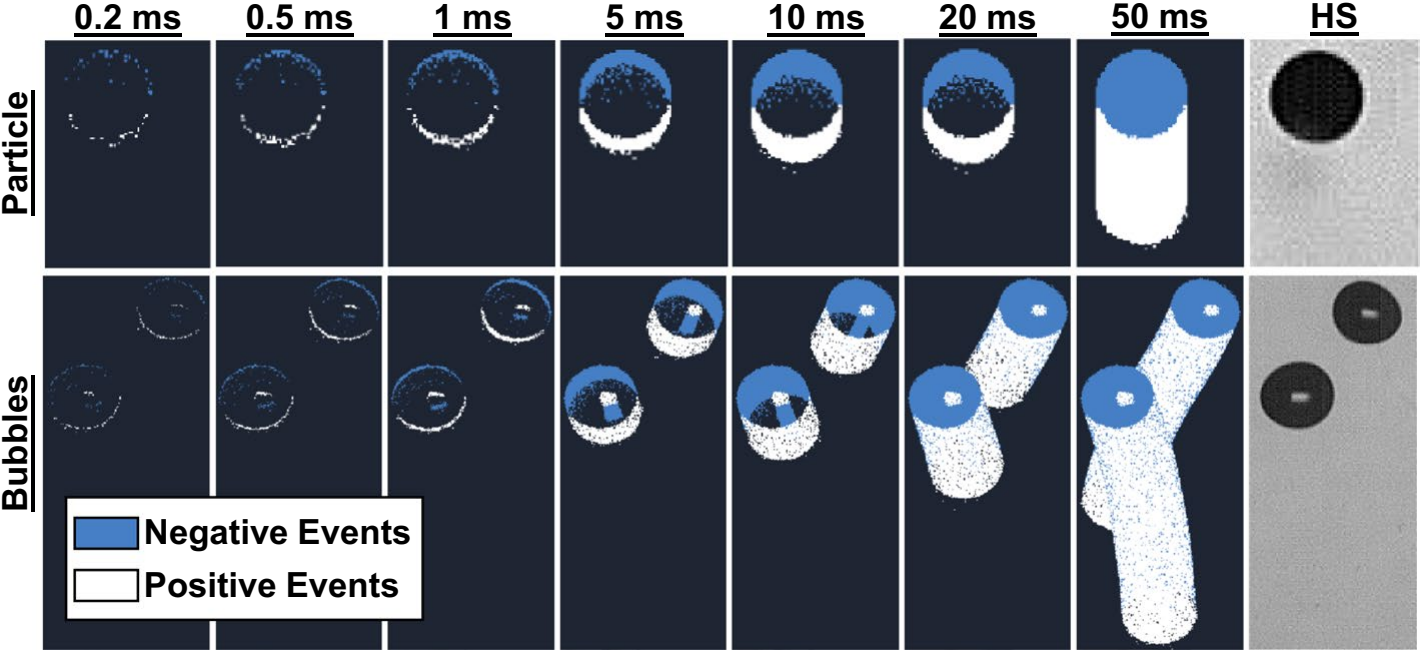
The synchronized output of the EVS camera for different accumulation times compared to the HS camera image, capturing a particle (top row) and two bubbles (bottom row)

Since the EVS camera continuously captures the events, a discrete frame at time *T* can be generated by accumulating the acquired events between time T and *T-*d*t*, where d*t* refers to the duration of the accumulation period. Increasing the accumulation time (d*t*) allows the camera to gather and display more events leading up to the frame time. As highlighted in Fig. 3, setting an appropriate accumulation time is necessary to allow the EVS camera to gather enough events to represent the particle or bubble shadows (coloured in blue) (Mueggler et al. 2015). The minimum accumulation time required for a full representation can be approximated by the ratio of their size (in pixels) to their velocity (in pixels per second). As particles or bubbles move, they cease to block light at their previous positions, leading to an increase in light intensity and the generation of positive events (shown in white). These positive events represent the tracks of the particles/bubbles, and the longer the accumulation rate, the longer the tracks. These tracks from the EVS camera are unique and useful for extracting their velocities and path information, as will be discussed later. Since the tracks represent the history of the bubble’s path, one can potentially identify whether there is a visual overlap in the camera view between bubbles in the past using the current instantaneous image, as can be seen in Fig. 3 (50 ms).

## Image processing

To segment the shadows of particles or bubbles, track pixels, and refine them within the frames generated by the event camera, the open-source imaging software FIJI (Schindelin et al. 2012) was used. Following this image processing, in-house MATLAB code was employed to extract quantities such as bubble size, velocity, and trajectory. The frames reconstructed from the event camera contain only pixels of one of three colours, namely positive, negative, and no events. This is advantageous for binarization, as it does not require thresholding, which is otherwise necessary for grey-scale images acquired from an HS camera. The accumulation time was increased post-recording to gather enough events to represent the entirety of the particles and bubbles, as can be observed in Fig. 3 at 50 ms for particles and 20 ms for bubbles. The appearance of holes within bubbles due to positive events from reflected light is mitigated by applying a hole-filling step during image segmentation. Following this process, the binary median filter was applied to remove noise in the background and at the boundaries of the particles/bubbles. The applied filter was limited to a 2 × 2 kernel to smooth the boundary of the particle without significantly altering the shape and dimension of the particles/bubbles. The segmentations of particles/bubbles in the images acquired from the HS camera adopt a similar technique for those with spherical or nearly spherical shapes. The dimensions and morphologies of the particle/bubble pixels were analysed using custom MATLAB code, which also utilized centroid tracking to estimate velocities. The Particle diameters are determined from the area ($$A)$$ of their segmented pixels, as $$D=\sqrt{4A/\pi }$$. To address the non-spherical shape of the bubbles, the bubble equivalent diameters $${(D}_{\mathrm{e}\mathrm{q}})$$ are calculated from the lengths of the major horizontal axis $${(D}_{\mathrm{h}})$$ and minor vertical axis $${(D}_{\mathrm{v}})$$ as $${D}_{\mathrm{e}\mathrm{q}}={{{(D}_{\mathrm{h}}}^{2}{D}_{\mathrm{v}})}^{1/3}$$, following the approach of Kim et al. (Kim et al. 2016).

However, as illustrated in Fig. 3, a minimum accumulation time (depending on its temporal displacement and size) is required to obtain a filled particle/bubble image. For instance, in the case of particles, the minimum accumulation time was 50 ms, which is greater than the interframe time of 1 ms, given a frame rate of 1000 Hz. Thus, for the particles recorded within t < 50 ms, a different processing strategy is needed to extract their size information.

**Fig. 4:**
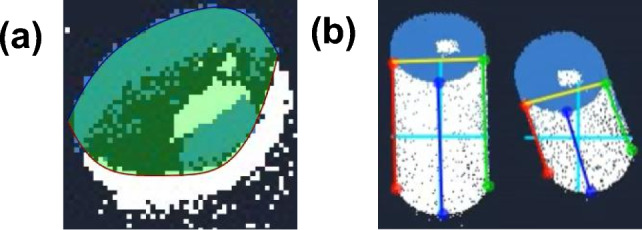
**a** The output of the in-house algorithms for curve fitting of the bubbles’ boundaries. **b** The pinpoints and lines that represent the track’s key information are extracted by in-house developed algorithms

To do this, a MATLAB code was developed to identify the area formed by the intersection of two fitted lines, as illustrated in Fig. 4 (a). The first line is fitted along the curvature of the top surface of the particles/bubbles, represented by negative events. The second line results from fitting the curvature of the top surface of their tracks. The boundary pixels are identified by using MATLAB’s convhull function, which internally uses the Quickhull algorithm (Barber et al. 1996). The usage of the technique is applied sparingly because of its susceptibility to noise along the boundary.

Finally, as discussed earlier, the tracks generated by the EVS camera can also be processed to estimate the size and velocity information of the particles/bubbles. For example, as illustrated in Fig. 4 (b), the width of the tracks matches the horizontal axis dimension of the particles/bubbles, and their length changes according to the accumulation time. To measure the length and width of these tracks, we start by dividing the tracks’ pixel region into four equal sub-regions, as shown by the light blue line in Fig. 4 (b). Subsequently, the longest distance from the track centroids is calculated to determine the track’s corner points. The dark blue line in Fig. 4 (b) represents the height of the track, which is measured by finding the midpoint along the top and bottom curvature of the track between the identified top and bottom corners. Short tracks resulting from a low accumulation time or slow movement setting can make it challenging to identify the bottom corners of the tracks, while extended tracks may exhibit significant curvature due to the lateral bubble motion, as illustrated in Fig. 3.

To limit the sources of inaccuracies, overlapping bubbles have been omitted from this analysis. However, the information obtained from analysing the pixels (positive/negative) of the bubbles, tracks, as well as their light reflections, could provide key information to identify overlapping bubbles in the camera view. This will be investigated in future work, where a multi-EVS camera setup will be used for benchmarking. Due to the three-color nature of the EVS camera, the overlapping analysis through an advanced algorithm could be very beneficial since it requires a much lower computational cost compared to conventional HS camera frames (Hessenkemper et al. 2022).

## Results and discussion

### Continuous illumination measurements

#### Particle release measurements

As stated in the experimental methodology section, the performance of the EVS camera and HS camera is compared in four experimental configurations. These configurations are based on the use of a particle chamber (polymer particles) or a compressed air diffuser (bubbles), and whether a continuous or pulsed light source was used for back illumination.

**Fig. 5:**
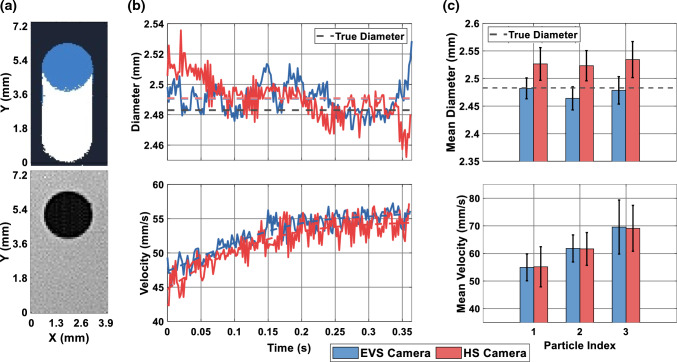
**a** Instantaneous images of a single particle captured by an EVS camera (top), HS camera (Bottom). **b** Variation of the particle’s measured diameter and velocity as it rises over time, as captured using both EVS and HS cameras. The black dashed line indicates the specified diameter of the particle by the manufacturer. **c** Mean diameter and velocity of all three subsequently released particles as measured by EVS and HS cameras

For the particle case, a total of three naturally buoyant polymer particles were released one after another. The image acquisition from both cameras began slightly before the particle release to ensure that all particles were captured within the measurement time window. As can be observed in Fig. 5 (b), the measured diameter of a single particle by the EVS camera is reported to be 2.4905 ± 0.0102 mm, which is almost identical to what is measured by the HS camera, 2.4909 ± 0.0132 mm. Both measurements are fairly accurate when compared to the stated particle size of 2.48 ± 0.004 mm by the particle’s manufacturer.

The slight difference in the measured particle size, as well as the fluctuation in measured diameter during the particle rise, can be attributed to several factors. These factors include scaling inaccuracies, pixel noise at the particle boundary, magnification effects, and particle movement along the depth. As can be observed in Fig. 5 (a), the particle shadow exhibits boundary noise in the EVS frame and blurring in HS frames, with shape inconsistencies across frames. These effects can be attributed to inhomogeneous illumination, optical disturbances from the liquid tank, and pixel-level artifacts caused by low magnification (EVS camera: 16.8 pixels/mm, HS camera: 9.83 pixels/mm). The presence of noise fluctuations at particle edges leads to inconsistent outputs from the smoothing algorithms, resulting in errors in the estimated bubble diameters. While using higher magnification could mitigate the effects of boundary noise, it was not implemented due to limitations in resolution and event-based saturation within the imaging system. Additionally, higher magnification was avoided to maintain consistent scaling, as the bubbles analysed in the following sections exhibit significant lateral motion, necessitating a larger field of view.

Furthermore, the estimated velocity also exhibits close agreement between both cameras (Fig. 5 (b)). The mean diameter and velocity of all particles (i.e., particles 1, 2, and 3) that were subsequently released are compared in Fig. 5 (c), differing by less than 2.4% and 0.65%, respectively. The similarity observed in the mean diameter across all particles highlights the repeatability of the measurements. The error bars in Fig. 5 (c) denote the standard deviations observed between each instantaneous image. The variations in the mean velocity across particles 1, 2, and 3 in Fig. 5 (c) is due to a change in their trajectories caused by interaction with the wake from the leading particle (Tavanashad and Subramaniam 2020). The measured time delay between successive particles is 270 ms between the first and second particle, and 204 ms between the second and third particle. The observed variation is also influenced by the manual opening of the particle chamber gate.

#### Bubbly flow measurements under low compressed air flow rate

**Fig. 6:**
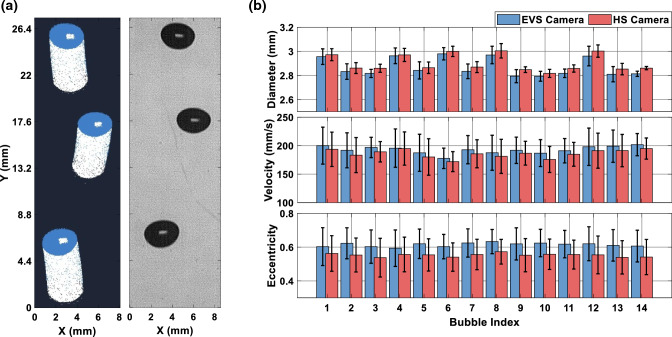
**a** Comparison of the instantaneous images simultaneously captured from EVS and HS cameras. **b** Measurement of fourteen different air bubbles released in water mixed with surfactants at low pressure, captured by EVS and HS cameras

Following the initial assessment and validation with the buoyant particles, the performance of the EVS camera in capturing bubbly flow is evaluated by analysing the dynamics of bubbles injected at the bottom of the tank using an air diffuser. Initial experiments (case I in Table 1) were conducted using a low volumetric flow rate of 6 ml/s and water mixed with surfactant at 25.9 mN/m to generate slow-moving, relatively spherical bubbles at a rate of 4.65 Hz, which enabled easier observation. As shown in Fig. 6 (b), the diameters and velocities measured for fourteen different bubbles by the EVS camera closely matched those obtained with the HS camera, with average differences of 0.5% for diameters and 3.5% for velocities. The error bars represent the standard deviation in the measured diameter and velocity during each bubble rise (Their motion can be observed in Supplementary Video 1). Similar to the particle rise case, the differences observed between the cameras stem from the magnification effects (EVS camera: 19.6 pixels/mm, HS camera: 12.7 pixels/mm), bubble boundary interface noise, and the influence of background conditions. Furthermore, the non-spherical shape of the bubbles contributes to measurement variation due to the slight difference in viewing angles between the cameras. These factors also influence the discrepancy in geometric measurements of the bubbles between the cameras. This discrepancy is more pronounced in eccentricity, with an average of 9.9%, potentially due to the flatter side edges captured by the EVS camera compared to the HS camera.

#### Influence of accumulation time

Among the key features of the EVS camera is its ability to adjust the accumulation time post-recording. Selecting a sufficiently long accumulation time to capture all events required to represent the bubble shadow enables the determination of their dimensions in pixel space. As outlined in the Experimental Methodology section, the accumulation time should be greater than the ratio of the bubble size to its velocity for the full representation. However, since bubbles undergo changes in size and velocity during their rise in the liquid, the selected accumulation time should account for the maximum bubble size and minimum velocity. Excessive accumulation times lead to elongated tracks and increased retention of noise events, as illustrated in Fig. 3. A reduced accumulation time is necessary when the total recording duration is shorter than the desired accumulation time. It may also be needed when short bubble tracks are preferred to avoid overlap due to the spatial proximity of the bubbles, while still retaining enough bubble shadow pixels to reliably determine bubble dimensions.

**Fig. 7:**
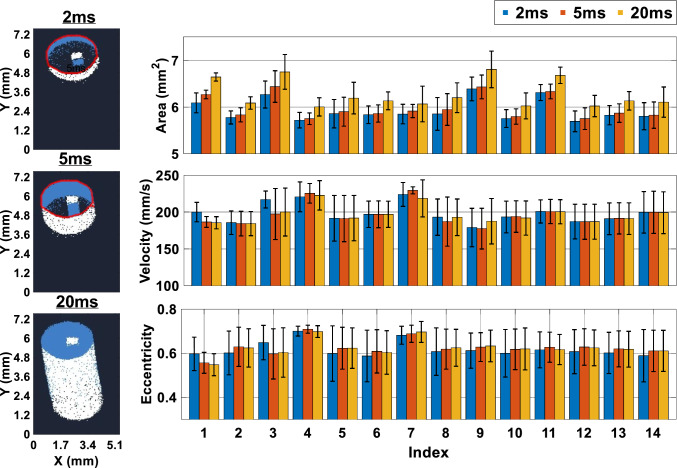
Air bubble measurements in water mixed with surfactants under low volumetric flow rate (case I in Table 1) for accumulation times of 2 ms, 5 ms, and 20 ms. The red lines in the 2 ms and 5 ms images show the bubble regions identified by the fitting algorithm, while the 20 ms case is measured directly from the bubble shadow (blue pixels)

As described in the image processing section, bubbles recorded with insufficient accumulation time for capturing all events generated by their shadows are sized using a boundary-fitting approach. Therefore, assessing the influence of accumulation time on the measured bubble size is necessary. The performance of the fitting technique for bubble measurement (Case I in Table 1) at accumulation times of 2 ms and 5 ms was compared against a reference case with sufficient shadow pixels, obtained using a 20 ms accumulation time. As illustrated in the plots of Fig. 7, the employed boundary fitting technique yields similar performance in measuring the velocities and eccentricity of the bubbles across different accumulation times, with average differences of 1.8% and 3.4%, respectively, relative to measurements based on all available bubble shadow pixels at 20 ms. However, the area identified through fitting is slightly smaller than when sufficient events are available to fully represent the bubbles’ shadows, particularly at lower accumulation times. The area measurements discrepancy arises due to missing boundary pixels, which can be as high as 8.5% at a dt of 2 ms and 5.6% at a dt of 5 ms when compared to a dt of 20 ms.

#### Velocity estimation from bubble tracks

**Fig. 8:**
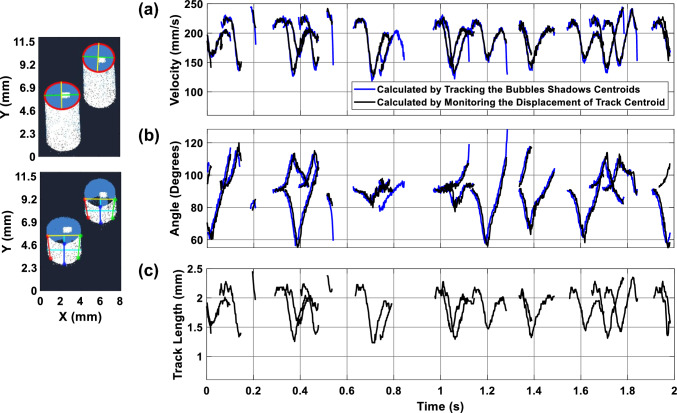
EVS camera calculated **a** velocities and **b** trajectories angles with time of air bubbles released in water mixed with surfactants at low volumetric flow rate by tracking the shadow of the bubbles’ centroids for an accumulation time of 20ms, and by monitoring the displacement of the tracks’ centroids for an accumulation time of 10ms. **c** The calculated track length of the bubbles versus time for an accumulation time of 10ms. Each connected line represents a different bubble evolution with time

The EVS camera’s ability to capture bubble tracks offers an additional advantage for characterizing bubbly flows. The plots in Fig. 8 (a) and (b) demonstrate that, for case I in Table 1 and at an accumulation time of 20 ms, measuring bubble velocity magnitudes and trajectory angles using the tracks’ centroids displacement yields results that closely match those obtained from the traditional approach of tracking the displacement of the bubble centroid. The average differences in bubble velocity and angle are below 0.71% and 0.43%, respectively. A closer examination of the particle and bubble tracks in Fig. 3 reveals that the particle track length is significantly shorter than that of the bubbles for all the accumulation times. This difference in the length of the tracks is attributed to their velocities. When comparing the track length to the velocity measured by its centroid shift (Fig. 8 (a) and (c)), a clear alignment is observed, distinguished mainly by a scaling difference. This scaling factor is the inverse of the accumulation time in seconds, indicating that the:

1 $$\begin{array}{c}\text{Track Velocity} \left(\mathrm{m}\mathrm{m}/\mathrm{s}\right)= \frac{\mathrm{T}\mathrm{r}\mathrm{a}\mathrm{c}\mathrm{k}~ \mathrm{L}\mathrm{e}\mathrm{n}\mathrm{g}\mathrm{t}\mathrm{h}~ \left(\mathrm{m}\mathrm{m}\right)}{\mathrm{A}\mathrm{c}\mathrm{c}\mathrm{u}\mathrm{m}\mathrm{u}\mathrm{l}\mathrm{a}\mathrm{t}\mathrm{i}\mathrm{o}\mathrm{n} ~\mathrm{T}\mathrm{i}\mathrm{m}\mathrm{e}~ \left(\mathrm{s}\right)}\end{array}$$

Measuring the length of the bubbles’ tracks provides a rapid approach to determining their velocities. These tracks inherently reveal the trajectories and velocities of the bubbles, offering a qualitative visualization without requiring further processing, which is not trivial with the images acquired from the HS camera. In the context of fluid dynamics, this capability of EVS is highly beneficial in realizing Lagrangian particle/bubble tracking. Moreover, the reduced data rate capability of EVS enables real-time visualization of bubble trajectories, whereas the high data rate associated with HS cameras limits this functionality.

Additionally, track widths can also be used to estimate the diameter of the bubble/particle. For example, determining the distance between the top-left and top-right corners of the tracks, as illustrated by the yellow line in Fig. 4 (b), provides a decent estimate of the bubbles’ width, which is the same as the spherical bubbles’ diameter. However, measuring the bubble height from the tracks proved challenging because pixelation causes the line between the top-left and top-right corners to fall below the bubble’s centreline. Consequently, accurately determining the dimensions of non-spherical bubbles, particularly elongated or irregularly shaped ones, is not accurate using the tracks alone.

**Fig. 9:**
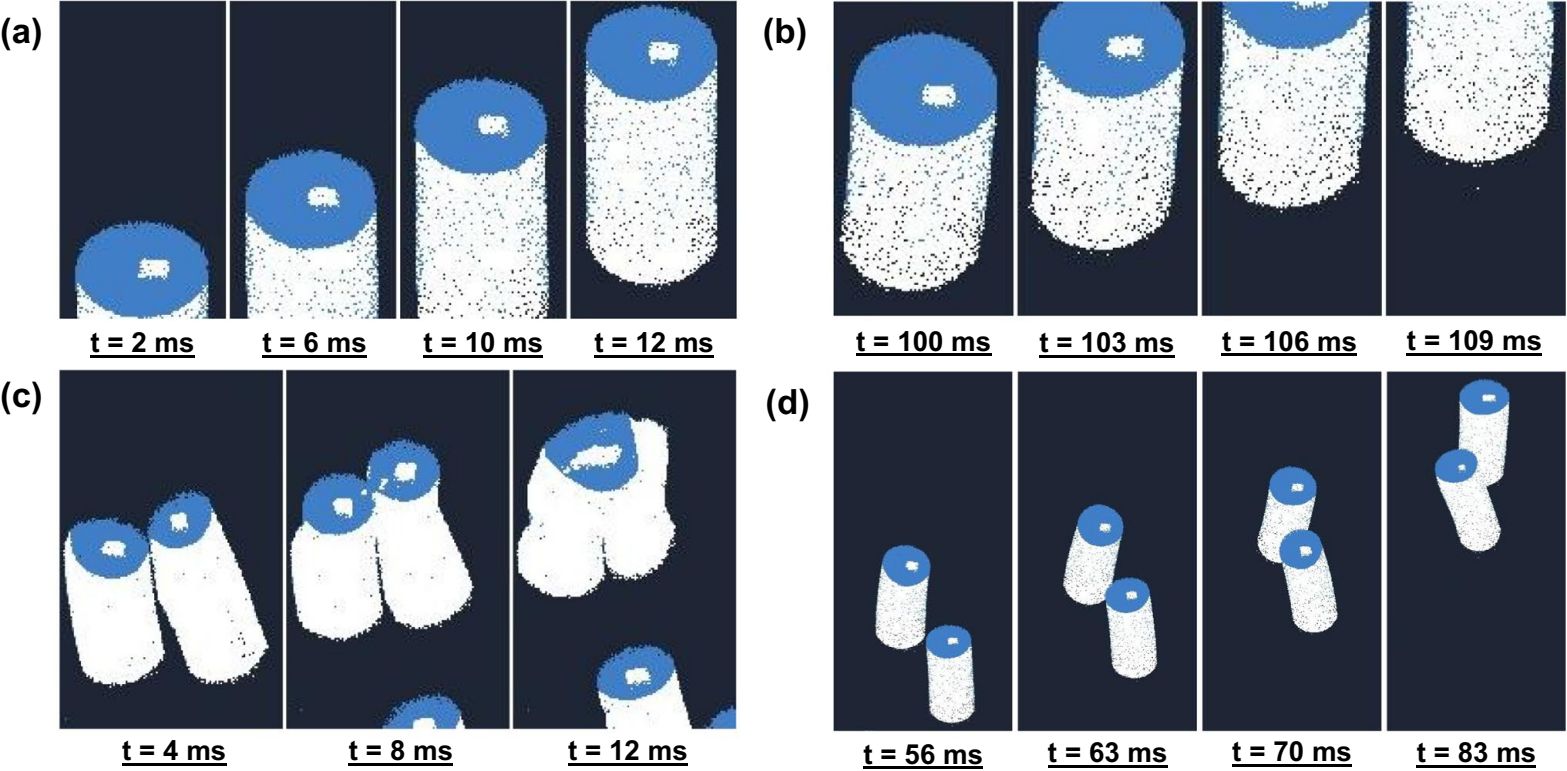
Sequences illustrating the advantages of using bubble tracks from EVS **a** Estimation of velocity using bubble shadows as the bubble enters the field of view. **b** Estimation of velocity using bubble tracks as the bubble leaves the field of view. **c** Visualization of bubbles’ coalescence, identified by the merging of individual bubble tracks. **d** Unsuitability of using tracks for measurement when an overlap occurs

Calculating velocities using a traditional approach by measuring the centroid’s displacement proves helpful as bubbles enter the frame or separate from overlapping conditions, as depicted in Fig. 9 (a; t = 2 ms). Similarly, velocity estimation based on bubble tracks is beneficial when bubbles are leaving the frame (Fig. 9 b; t = 106 ms) or interacting with other bubbles. Integrating both methods offers a more robust velocity estimation and helps recover data that would otherwise be lost in either approach alone, as shown in Fig. 8 (a). These tracks are also beneficial in monitoring the coalescence of bubbles or their visual overlap as observed in Fig. 9 (c). However, their utility is diminished when overlapping occurs with another bubble or track, as highlighted in Fig. 9 (d).

#### Bubbly flow measurements with elevated compressed air flow rate

**Fig. 10:**
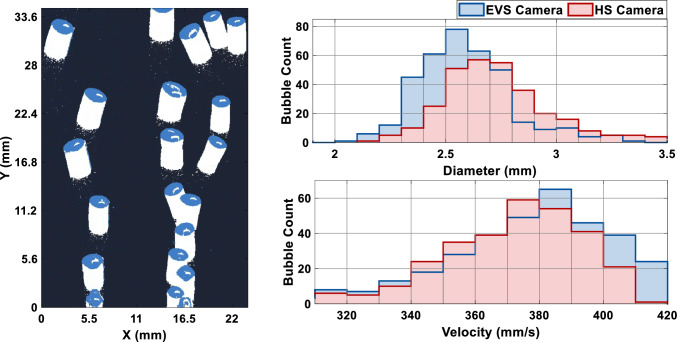
Size and velocity measurements of air bubbles released in water without surfactant at moderate compressed air flow captured by EVS and HS cameras

To further assess the EVS camera’s capability in capturing bubbly flow, the compressed air flow rate was increased to 75 ml/s, and no surfactant was added to the water. Under these conditions listed in Table 1 (case II), bubbles were generated at a higher rate of 100 Hz, moved more rapidly, and showed pronounced elongation along their sides. The histograms of bubble diameters and velocities measured by the EVS and HS cameras revealed trends consistent with those in the previous case, as shown in Fig. 10, where the EVS camera reports smaller diameters (by 5.9% on average) and slightly higher velocities (by 1.4% on average) than the HS camera. This trend is attributed to the loss of bubble shadow events at the interface, resulting from reduced probabilistic triggering induced by refractive effects.

#### EVS camera oversaturation

As stated in the introduction, EVS cameras have a finite design threshold for handling event rate, beyond which event oversaturation occurs. The event rate is defined as the rate of pixel activation in the EVS sensor, accounting for both positive and negative events, expressed in million events per second (*ME/S*). The event rate caused by the presence of bubbles and their motion under continuous illumination (${\mathrm{E}\mathrm{R}}_{\mathrm{c}\mathrm{o}\mathrm{n}\mathrm{t}\mathrm{i}\mathrm{n}\mathrm{u}\mathrm{o}\mathrm{u}\mathrm{s}})$ can be estimated as:

2 $$\begin{array}{c}{\mathrm{E}\mathrm{R}}_{\mathrm{c}\mathrm{o}\mathrm{n}\mathrm{t}\mathrm{i}\mathrm{n}\mathrm{u}\mathrm{o}\mathrm{u}\mathrm{s}}\approx \sum_{i=1}^{n}\left[\left({A}_{px, i}+ {T}_{px, i}\right)\bullet {f}_{a,i}\right]\end{array}$$

where $n$ is the number of bubbles present within the Region of Interest (ROI), ${A}_{px, i}$ is the pixel count of the area of the bubble $i$, ${T}_{px, i}$ is the pixel count of the area of the bubble $i$ track, and ${f}_{a,i}$ is the frequency (Hz) at which the $i$ ^th^ bubble-track pair motion induces pixel activations in the EVS camera sensor, and it is proportional to the pair velocity. The use of the approximation symbol in Eq. (2) reflects that the model does not account for secondary factors, including background noise and variations in contrast sensitivity bias.

**Fig. 11:**
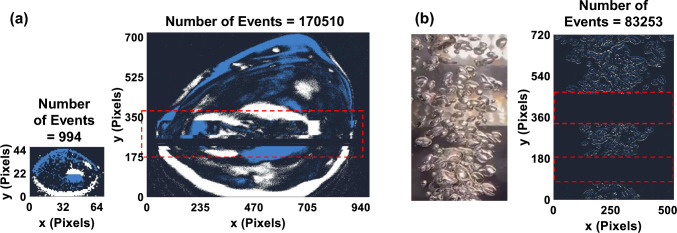
The negative effects of event oversaturation on the EVS camera recording. **a** Pixel desynchronization, seen as misaligned bubble pixel rows, occurs during high-magnification (230 pixels/mm) imaging of fast bubbles. **b** Temporal loss of events at high bubble density, marked by the red cross-section

When oversaturated, the camera may exhibit several undesirable effects during bubble imaging, including inaccurate timestamps, missing events, footage distortion (observed in Supplementary Video 2), and temporary data blackouts. Event saturation becomes more pronounced under high magnification, as the number of events generated per bubble increases proportionally with magnification (Fig. 11 (a) shows a comparison between 21.1 pixels/mm and 230 pixels/mm. Under high magnifications, event oversaturation can cause distortions manifested as desynchronization and loss of bubble pixel rows. This effect arises from delays in the EVS row arbiter event registration and timestamping process, as seen in Fig. 11 (a)Fig. 11. Increasing the compressed air flow rate via the diffuser can also induce oversaturation even at relatively low magnification (21.1 pixels/mm), as it produces a dense field of rapidly ascending bubbles, triggering events across too many pixels. This increases the number of events (event rate), causes the row arbiter to oversaturate and lag in processing and timestamping the events. Consequently, the temporal loss of events occurs, as can be observed in Fig. 11 (b).

The timing precision of the EVS camera is adversely affected by sensor latency, which can cause discrepancies between the actual occurrence and the recorded timing of events. To measure the impact of oversaturation-related timing errors, the light source was turned off when oversaturation was detected during recording. The EVS camera continued to report events post-darkness, demonstrating significant readout latency issues.

**Fig. 12:**
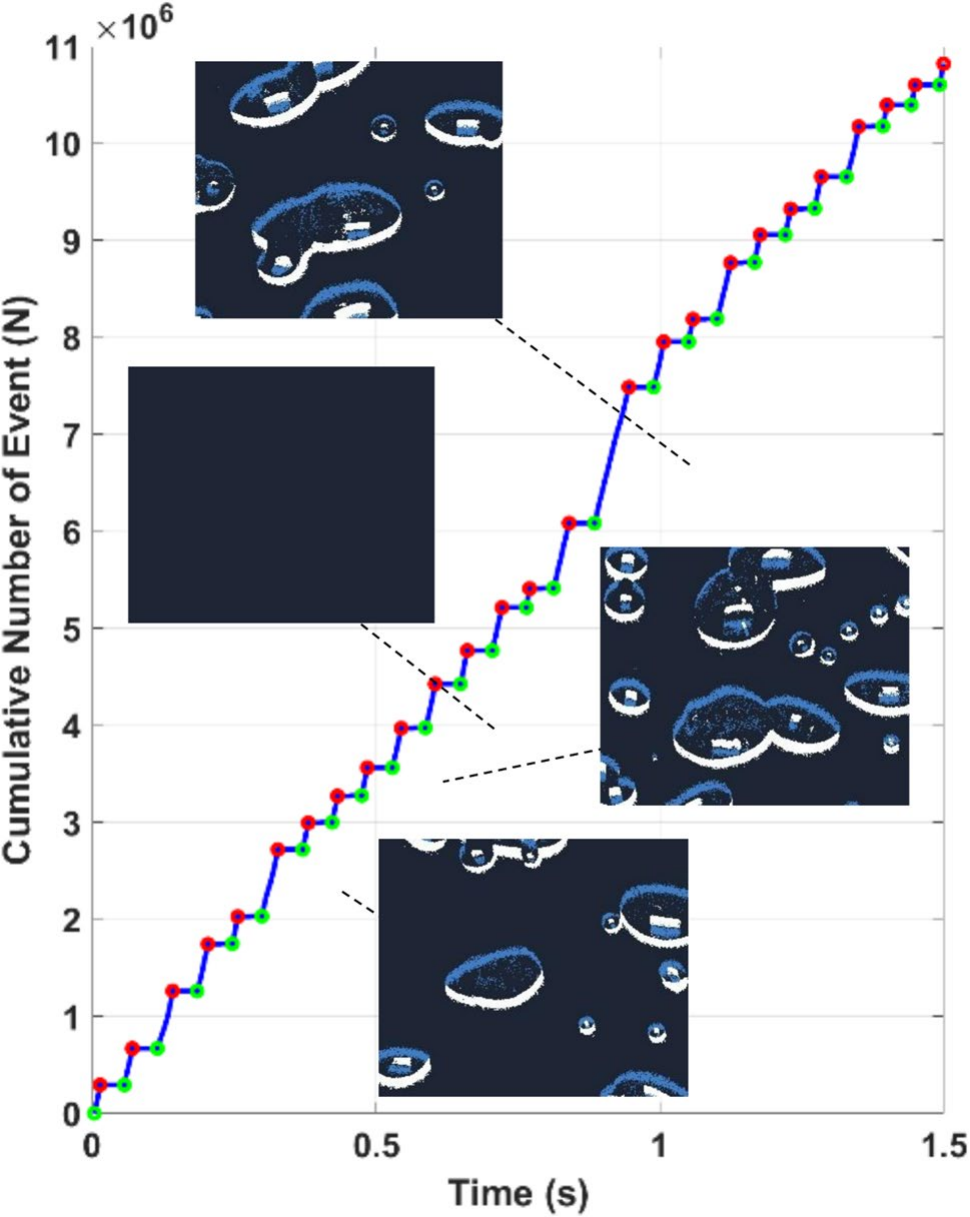
Cumulative number of events recorded by the EVS camera under intermittent periods of complete readout loss versus time. The superimposed images on the graph highlight the difference in event rate between and during blackouts. Horizontal lines represent readout losses

Under conditions of a reduced ROI of 300 × 250 pixels, and a magnification of 21.1 pixels/mm, and a relatively high volumetric flow rate of 200 ml/s (case III in Table 1), the EVS camera occasionally produced frames with intermittent periods of complete readout loss, as can be observed in Supplementary Video 3. In Fig. 12, the cumulative number of events is plotted over time, clearly indicating periods of readout loss as indicated by the horizontal lines in the curve. The duration of these periods was consistent at nearly 43 ms. It can be observed that more events are detected over longer durations when the event rate is slower before readout loss occurs. However, this trend is not consistent or directly proportional, which further complicates the appropriate selection of ROI size to avoid the occurrence of this problem.

### Pulsed illumination measurements

**Fig. 13:**
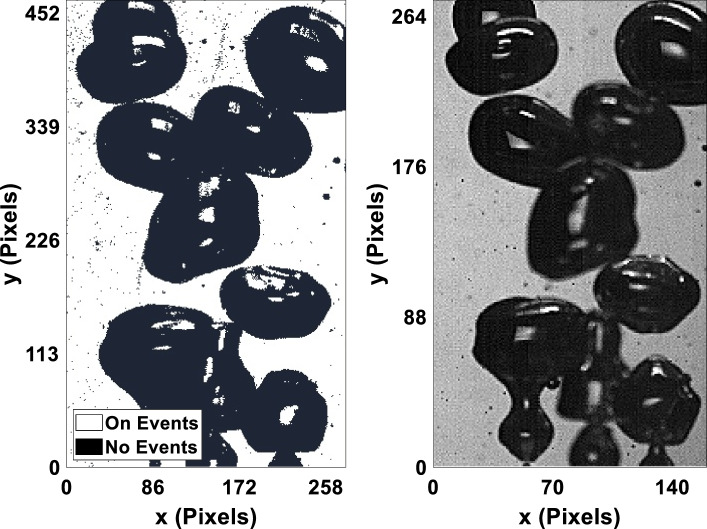
Dense bubbly flow under using pulsed illumination recorded with EVS camera (left), and HS camera (right). The white pixels reflect the number of events captured by the EVS camera

The reliable capture of dense and fast-moving bubbly flows with the current EVS cameras necessitates constraints on ROI and magnification. However, implementing pulsed illumination may enable successful imaging under these conditions while allowing for a larger ROI, such as Case IV in Table 1, shown in Fig. 13 and Supplementary Video 4. Under pulsed illumination, the light pulse induces a rapid, uniform change in background intensity that is registered as events. The resulting event rate under pulsed illumination (${\mathrm{E}\mathrm{R}}_{\mathrm{p}\mathrm{u}\mathrm{l}\mathrm{s}\mathrm{e}\mathrm{d}}$) is primarily governed by the pulsed background illumination within the ROI, not obscured by bubbles. By excluding the pixel count of the bubble reflection region $({R}_{px, i})$, this event rate can be estimated as a function of the pulse frequency (${f}_{\mathrm{p}\mathrm{u}\mathrm{l}\mathrm{s}\mathrm{e}}$) in Hz as follows:

3 $$\begin{array}{c}{\mathrm{E}\mathrm{R}}_{\mathrm{p}\mathrm{u}\mathrm{l}\mathrm{s}\mathrm{e}\mathrm{d}}\approx [ROI- \sum_{i=1}^{n}\left[\left({A}_{px, i}- {R}_{px, i}\right)\right]\bullet {f}_{\mathrm{p}\mathrm{u}\mathrm{l}\mathrm{s}\mathrm{e}}\end{array}$$

**Fig. 14:**
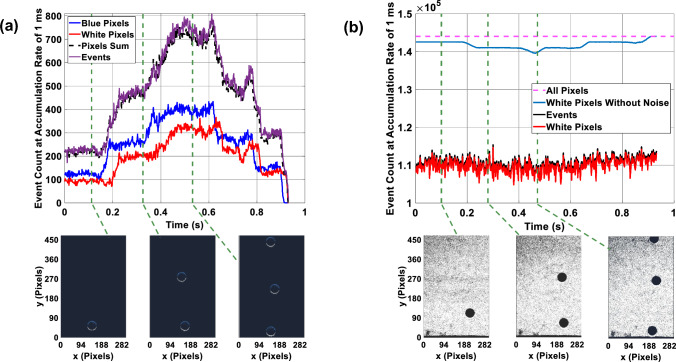
The number of events resulted from the release and movement of three particles with a 1-ms accumulation time under **a** continuous illumination, **b** pulsed illumination. Blue pixels correspond to negative events, while white pixels correspond to positive events

To demonstrate the difference between pulsed and continuous illumination on the EVS camera event generation, the number of events produced by three particles moving through the water tank for an accumulation time of 1 ms is compared. As can be observed in Fig. 14 (a), under continuous illumination, the measured number of events increases as the number of particles increases within the field of view of the EVS camera. The number of events peaks when (t ~ 0.575 s) all the particles are within the field of view. Counting the number of blue and white pixels, corresponding to negative and positive events, respectively, does not directly reflect the actual number of events, due to the overlap of events within the accumulation time window.

On the other hand, as illustrated in Fig. 14 (b), in the absence of particles and noise, the number of events generated by pulsing the light closely matches the number of pixels in the chosen ROI, as pulsed illumination induces intensity changes across all the exposed pixels. When the particle is introduced in the ROI, a reduction in the number of events caused by the particles occurs, which directly matches the collective number of their pixels based on their presence within the frame and their diameters. This highlights the unique fact that the increase in particles or bubbles within the ROI will lead to a decrease in the number of events. This contrasts with the continuous illumination, where the number of events increases with the increase in the number of particles/bubbles. Therefore, the utilization of a pulsed illumination would be advantageous in the situation where the particles or bubbles occupy a large portion of the ROI, whether due to their size or their number. It should be noted that, as all the exposed pixels (except those blocked by particles) generate events during pulsed illumination, this necessitates limiting the light pulsing frequency and pulse duration.

Beyond total event count, the mechanisms of event generation differ fundamentally between illumination methods. Under continuous illumination, the event rate scales with object velocity. Within a fixed accumulation window, fast-moving bubbles generate dense events along their boundaries, whereas slow-moving bubbles produce fewer events per unit time. Consequently, slow bubbles require longer accumulation times to achieve a fully closed boundary, as observed in Fig. 3. This introduces an inherent velocity-dependent bias and complicates the accumulation time selection. Pulsed illumination removes this dependency by shifting the triggering mechanism from object motion to the light source. A short light pulse induces an instantaneous brightness change across the field of view, generating events along the full contour of each bubble simultaneously, whether fast, slow, or stationary. The selected accumulation time window under pulsed illumination must match the pulse frequency; a window that is too short yields empty frames that miss the pulse entirely, whereas an excessively long window merges multiple pulses into a single frame.

The data rate of the EVS camera is another key consideration. As described in the Introduction, the camera records events only when illumination changes. In Fig. 14 (a), under continuous illumination, events correspond solely to the particles’ presence in the ROI, and the total data volume increases with the number of particles in the ROI, as summarized in Table 2**.** Under pulsed illumination, the particles occupy only a small portion of the ROI, so their presence does not significantly affect the data rate. For scenarios where particles or bubbles occupy a larger fraction of the ROI, their presence and number can influence the data rate and event rate more significantly. For example, in the case shown in Fig. 13, the data rate fluctuates between 30.6 MB/s and 45.8 MB/s, and the event rate fluctuates between 5.72 ME/s and 8.59 ME/s. The varying relationship between event rate and data rate reflects the dynamic compression efficiency of the EVS camera’s native EVT3 encoding format (Aitsam et al. 2024). While the relatively sparse event streams generated under continuous illumination result in lower compression efficiency, the dense bursts of uniform-polarity events produced under pulsed illumination maximize row-vectorized encoding. This highly correlated event stream enables efficient hardware packet sharing, substantially reducing the resulting data footprint.

**Table 2:** The average data rate and event rate of capturing the rise of three particles using the EVS camera and the HS camera

	One particle		Two particles		Three particles		Overall Particles Raise	
	Data Rate (MB/s)	Event Rate (ME/s)	Data Rate (MB/s)	Event Rate (ME/s)	Data Rate (MB/s)	Event Rate (ME/s)	Data Rate (MB/s)	Event Rate (ME/s)
EVS Camera Continuous Illumination	1.43	0.225	2.68	0.471	3.92	0.729	2.54	0.447
EVS Camera Pulsed Illumination	34.1	111	34.0	109	33.9	109	34.0	110
HS camera	199	–	199	–	199	–	199	–

Although pulsed illumination generates frames that resemble conventional HS camera images, the data rate remains substantially lower due to the binary representation of events.

Unlike continuous illumination, the latency does not result in mistiming since all accumulated events correspond to a single pulse. This behaviour was thoroughly documented by the investigation on particle imaging velocimetry (PIV) using pulsed illumination (Willert 2023). Furthermore, accurate timestamping is necessary for measurements in high-speed flows and sudden acceleration in even low-velocity flows. In these situations, pulsed illumination is advantageous for accurate event timestamping. However, the use of pulsed illumination negates the EVS camera’s unique ability to continuously capture events and generate tracks representing particle and bubble motion.

**Fig. 15:**
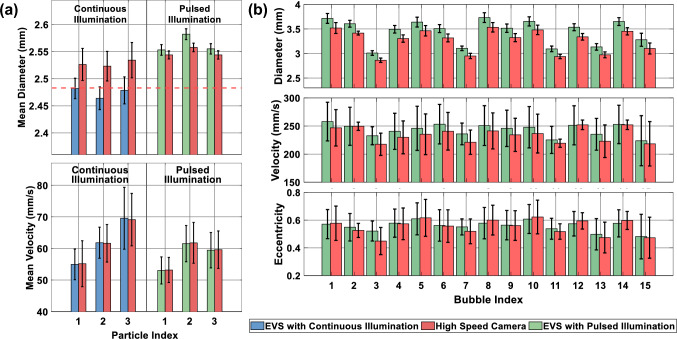
Comparison of the EVS camera under pulsed illumination to the HS camera for measuring: **a** the diameters and velocities of 3 precisely manufactured particles, and **b** the diameters and velocities of bubbles generated under low volumetric flow rate conditions in a tank filled with surfactant-mixed water (which leads to nearly spherical bubbles)

Capturing particle release with pulsed illumination using the EVS camera was also performed simultaneously with the HS camera to benchmark its measurements. As shown in Fig. 15, the results obtained from the EVS camera closely match those of the HS camera, with measurement differences of less than 0.40% in particle velocities and 0.95% in particle diameters under pulsed illumination. The difference in the particles’ velocities between the continuous and pulsed illumination cases can be attributed to the interval duration between the particle release (successive particles were 270 ms and 204 ms in the continuous-illumination experiment; in the pulsed-illumination experiment, they were 242 ms and 228 ms). The average measurement differences for bubbles were 5.4% in diameter, 3.7% in velocity, and 3.5% in eccentricity. The measured particle diameter difference (< 3%) between continuous and pulsed illumination is due to differences in edge characteristics. Continuous illumination produces weaker brightness gradients and fewer edge events, yielding slightly smaller diameters, while pulsed illumination generates sharp intensity transitions along the bubble boundary, producing more events and slightly larger diameters. Overall, these results indicate that there is no significant deviation from measurements acquired under continuous illumination.

While the EVS camera’s readout latency does not affect event timing accuracy during pulsed illumination, triggering events across all exposed pixels increases the event rate, which can eventually lead to oversaturation. This oversaturation could be limited by decreasing the light pulsing frequency and pulse duration, as each pulse generates many events. It is also important to limit the number of activated pixels. The most reliable strategy is to reduce the ROI, as activating all pixels within a small ROI during each pulse will not result in oversaturation unless an extreme pulse rate is used. Oversaturation can also be mitigated if a significant portion of the ROI is obstructed by particles or bubbles (e.g., dense bubbly flows). However, any unexpected reduction in their presence (e.g., transient operation) during the imaging may increase the number of events and the risk of oversaturation. During pulsed illumination, since the EVS camera generates events discretely, it does not produce any tracks, unlike during continuous illumination.

## Operational advantages and limitations and recommendations for measuring bubbly flows with EVS cameras

As demonstrated in this assessment, the EVS camera is capable of characterizing bubbly flows with accuracy and sampling speed comparable to that of a frame-based HS camera. Furthermore, it offers some unique advantages, including the ability to record only bubbles, excluding the stationary background during continuous illumination. This feature eases the efforts required for background removal in the raw images in situ. The acquired event stream output consists only of positive and negative events, which significantly reduces the data rate and eventually storage requirements. This feature of EVS enables real-time bubble imaging even at a higher sampling rate. Whereas a high data rate associated with an HS camera limits its real-time imaging capability. Furthermore, as shown in Fig. 8, the continuous capturing of events enables the generation of tracks that match the bubble velocity, enabling bubble trajectory visualization.

**Fig. 16:**
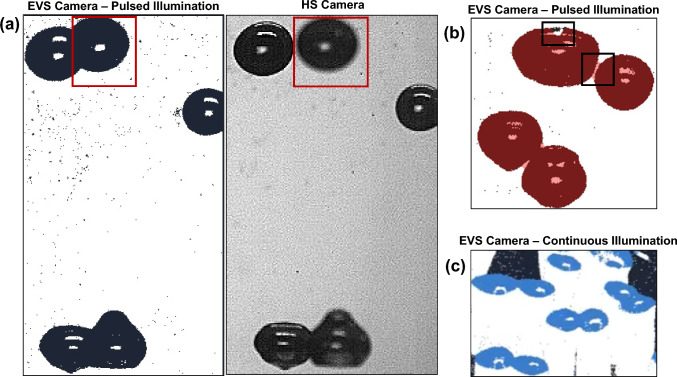
Visual challenges of the EV camera for characterizing bubble flow. **a** Lack of Depth information. **b** Binary segmentation. **c** Tracks overlapping

However, the EVS camera is not without limitations. Since the EVS camera captures only positive and negative events, along with the absence of events, its visual output is fundamentally restricted to three distinguishable colours. This limits the application of the gradient function to estimate the magnitude of sharpness/blurriness in the image. Furthermore, because the EVS camera is very sensitive to light, it necessitates a large *f-number* setting (small aperture) on the lens to avoid any random noise induced by ambient light. This limitation prevents the EVS camera from conveying depth information, as seen in Fig. 16 (a), where out-of-focus bubbles appear blurry on the HS image, whereas they appear focused on the EVS image. This can be mitigated by fitting a neutral density filter (NDF) to the EVS camera lens, allowing for the use of large aperture settings.

Next, the strong reflectivity of bubble surfaces poses challenges for accurate boundary detection by the EVS camera. High-intensity reflections cannot be effectively suppressed through light bias adjustment without introducing significant noise or omitting portions of the bubble. Binary processing algorithms have shown potential in mitigating this limitation. In many instances, they reduce boundary noise and enhance the accuracy of bubble centroid determination. However, in other instances, binary processing resulted in the merging of unconnected or non-overlapping bubbles, as well as the removal of portions of individual bubbles, as depicted in Fig. 16 (b). In cases of limited overlap, techniques such as the watershed algorithm may assist in separating merged bubbles. Nonetheless, identifying overlapping regions through boundary analysis is difficult when bubbles exhibit non-uniform or irregular shapes. Finally, while bubble tracks from EVS offer valuable insights into bubble velocities and trajectories, their utility diminishes at high bubble densities due to overlapping, as demonstrated in Fig. 16 (c). Moreover, as mentioned previously, they are entirely absent when pulsed illumination is employed.

The primary limitation hindering the performance of the EVS camera remains event oversaturation, which leads to several detrimental effects, including missed events, recording failures, inaccurate timestamps, and temporary blackouts, as previously discussed. Mitigating event oversaturation requires controlling the factors in Table 3 that influence the variables in Equations 2, 3. Under continuous illumination, both the bubble density and their velocities contribute significantly to the increase in event rates. In contrast, under pulsed illumination, bubbles act as light obstructions, reducing the number of events, while their velocities do not influence event generation. However, the pulse rate must be sufficiently high to capture temporally resolved bubble motion, which in turn can contribute to event oversaturation. The light reflected by the bubbles will cause the generation of events in both continuous and pulsed illumination. The use of light diffusers and blocking ambient light sources could help limit their presence. While magnification enhances light obstruction by bubbles under pulsed illumination, it poses a significant challenge under continuous illumination, where even a single large bubble can induce oversaturation as it triggers events across too many pixels. The selection of an appropriate magnification is critical for EVS-based measurements to accurately determine bubble size from the spatial distribution of events generated by the bubble’s shadow. Under continuous illumination, when the bubble’s representation in pixel space is low, the impact of missing events (arising from refractory effects or probabilistic triggering) and background events increases, and noise becomes increasingly significant, leading to larger centroid estimation errors. Furthermore, the event triggering mechanism directly influences the measured bubble size, as illustrated in Fig. 15. Under pulsed illumination, the measured diameters are slightly larger and more consistent with the HS camera results due to the EVS camera’s strong response to the overall pulsed illumination change, producing thicker or more pronounced edges, In contrast, under continuous illumination, the measurements are based on the bubble motion and more susceptible to probabilistic triggering effects, resulting in less consistent edge definition. Although managing the aforementioned factors can mitigate oversaturation, abrupt changes in bubble count or velocity may still cause event saturation. Consequently, restricting ROI is critical to maintaining data reliability in complex bubbly flow scenarios. Determining an optimal ROI and magnification for measuring bubbly flow under continuous illumination is very challenging due to the dynamic nature of the bubble dimensions, motion, and number. In contrast, under pulsed illumination, it is possible to determine an ROI safe limit based on the extreme case where the pulsed illumination is entirely unobstructed by bubbles. This limit is defined as:

**Table 3:** Contributing factors to EVS camera oversaturation under continuous and pulsed Illumination conditions. Symbols indicate effects on oversaturation: “–” = reduction, “ + ” = increase, “/” = no effect

	Bubble counts	Bubble Velocities	Bubbles Light Reflectivity	Magnification	ROI	Pulsed Rate
Continuous illumination	** + **	** + **	** + **	** + **	** + **	**/**
Pulsed illumination	**-**	**/**	** + **	**-**	** + **	** + **

4 $$\begin{array}{c}{\mathrm{R}\mathrm{O}\mathrm{I}}_{\mathrm{l}\mathrm{i}\mathrm{m}\mathrm{i}\mathrm{t}}\left(px\right)< \frac{\mathrm{E}\mathrm{V}\mathrm{S}~ \mathrm{C}\mathrm{a}\mathrm{m}\mathrm{e}\mathrm{r}\mathrm{a}~ \mathrm{O}\mathrm{v}\mathrm{e}\mathrm{r}\mathrm{s}\mathrm{a}\mathrm{t}\mathrm{u}\mathrm{r}\mathrm{a}\mathrm{t}\mathrm{i}\mathrm{o}\mathrm{n} ~\mathrm{L}\mathrm{i}\mathrm{m}\mathrm{i}\mathrm{t}~ (\mathrm{M}\mathrm{E}/\mathrm{s})}{\mathrm{P}\mathrm{u}\mathrm{l}\mathrm{s}\mathrm{e} ~\mathrm{R}\mathrm{a}\mathrm{t}\mathrm{e} ~\left(1/\mathrm{s}\right)}\end{array}$$

Advances in EVS camera sensor development may lead to higher event saturation thresholds and lower latency, thus strengthening the camera’s performance and applicability in measuring complex and rapid bubbly flows.

## Conclusions

This study demonstrates the viability and limitations of EVS cameras for high-speed imaging of bubbly flows, with an emphasis on simultaneous bubble sizing and velocimetry. Noticeably, experiments were performed simultaneously with EVS and a conventional HS camera to obtain detailed performance metrics. The use of precisely manufactured and naturally buoyant particles as a benchmark reference confirms the EVS camera’s ability to deliver precise and consistent measurement results differing by a marginal 2.4% in measured diameters and 0.65% in measured velocities. Under continuous illumination conditions, the EVS camera demonstrated comparable accuracy to the HS camera in capturing both bubble dimensions and velocities with an average deviation of 0.5% for diameters and 3.5% for velocities. Furthermore, we demonstrated the novel approach of exploiting the bubble tracks offered by EVS to estimate the velocity in certain critical conditions, which is otherwise not possible with a conventional HS camera.

While EVS cameras offer substantial benefits such as reduced data rates, background-free imaging, and asynchronous event recording, their susceptibility to event oversaturation, particularly under continuous illumination, remains a key challenge. Factors such as bubble count, velocity, magnification, and ROI size significantly influence the risk of oversaturation. Strategic control of these parameters, particularly through ROI reduction and lighting selection, is essential for maintaining data reliability.

By applying pulsed illumination, event saturation and latency were successfully minimized in scenarios involving dense bubble populations and high magnification, permitting the EVS camera to measure diameter and velocity with good agreement to those of conventional HS systems. This approach, however, limits the EVS camera’s inherent advantage of continuous event acquisition and trajectory tracking.

Moreover, limitations associated with the bubbles’ surface reflectivity, depth perception, and event merging in dense flows were observed, especially under challenging optical conditions. Binary processing techniques and segmentation algorithms show partial success in mitigating these issues but require further refinement.

Fully resolving bubble morphology, dynamic shape fluctuations, and three-dimensional motion, particularly in the presence of bubble overlap, remains challenging with a single EVS or conventional camera. Previous research has successfully utilized multiple conventional cameras to overcome this limitation. Future work will examine the use of a multi-EVS camera system, address calibration challenges, and evaluate integrated measurements.

Advancements in EVS camera hardware, particularly in reducing sensor latency and increasing event saturation thresholds, are expected to extend their applicability in complex multiphase flow environments (e.g. dense bubbly flows). With these improvements, EVS cameras may become a more robust and versatile tool for the accurate and efficient characterization of bubbly flows. Finally, unlike conventional high-speed cameras, EVS cameras involve much lower data rates, allowing for real-time high-speed imaging. Real-time capability enables the implementation of automated imaging and decision-making software linked with AI. This, for example, allows system learning for real-time controls in transient applications like bubble dynamics near critical current density in electrolysis.

## Supplementary Information

Below is the link to the electronic supplementary material.

Supplementary file1 (MP4 21436 kb)

Supplementary file2 (MP4 275 kb)

Supplementary file3 (MP4 3245 kb)

Supplementary file4 (MP4 11363 kb)

## Data Availability

No datasets were generated or analysed during the current study.
